# Factors affecting menstrual hygiene among women living in slum areas of Budanilakantha Municipality, Kathmandu

**DOI:** 10.1371/journal.pone.0336321

**Published:** 2026-08-07

**Authors:** Bijaya Laxmi Niraula, Akshaya Acharya, Manoj Phuyal, Baburam Marasini, Puspa Acharya

**Affiliations:** 1 Department of Public Health, Nobel College, Pokhara University, Kathmandu, Nepal; 2 Central Department of Public Health, Institute of Medicine, Kathmandu, Nepal; 3 Department of Public Health, Nepalese Army Institute of Health Science, Kathmandu, Nepal; Cranfield University, UNITED KINGDOM OF GREAT BRITAIN AND NORTHERN IRELAND

## Abstract

**Background:**

Menstruation is often stigmatized and poorly managed in low-income settings like Nepal, leading to health and social challenges. Women in urban slums face additional barriers due to poverty and limited sanitation. This study explores factors affecting menstrual hygiene practices among women in Budanilakantha slums to inform targeted interventions.

**Methods:**

A cross-sectional study was conducted among 247 women aged 18–49 years in slum areas of Budanilakantha Municipality, Nepal. Participants were selected using systematic random sampling. Data were collected via a pretested questionnaire and analysed with chi-square tests. Ethical approval was obtained from Nobel College, Pokhara University.

**Results:**

Among the 247 women surveyed, 53.8% used sanitary pads and 42.9% used reusable cloth materials (traditionally used fabric pieces) during menstruation. Most participants reported using soap and water for handwashing (66.8%) and external genital cleaning (82.6%), whereas only 38.9% bathed daily during menstruation. Nearly all participants (95.1%) experienced menstrual restrictions, and 57.1% reported being perceived as impure. Statistically significant associations were observed between menstrual hygiene-related awareness and both education and monthly income, while handwashing practice was significantly associated with ethnicity, occupation, and monthly income. These findings suggest that socio-economic conditions and cultural norms are key determinants of menstrual hygiene practices among women living in slum areas.

**Conclusions:**

Menstrual hygiene practices in Budanilakantha slum areas are influenced by socio-economic status, cultural taboos, and limited infrastructure. Although basic hygiene is practiced by many, cultural stigma and gaps in hygiene behaviours persist. Comprehensive interventions addressing education, water, sanitation and hygiene infrastructure, and social norms are essential to improve menstrual health and dignity among marginalized women.

## Introduction

Menstruation is a natural biological process marking the transition from adolescence to womanhood. Despite its physiological normalcy, menstruation remains a subject of silence, stigma, and discrimination in many societies, particularly in low- and middle-income countries (LMICs) such as Nepal. Inadequate menstrual hygiene management (MHM) has been associated with negative health outcomes, including reproductive tract infections, urinary tract infections, and psychological distress. Moreover, it affects educational attainment, mobility, and the overall well-being of adolescent girls and women [1–2].

Globally, more than 500 million women and girls are estimated to lack access to adequate menstrual hygiene facilities and products [3]. The challenge is even greater in urban slums where poverty, poor sanitation, limited water supply, and deep-rooted cultural taboos hinder proper menstrual hygiene practices [4]. In Nepal, although national efforts have been made to address adolescent health, menstrual health is still not fully integrated into major public health agendas and remains under-addressed in policy and programme implementation [5]. Menstruation remains shrouded in silence and stigma in many low- and middle-income countries, where myths, misconceptions, and social restrictions continue to shape women's and girls’ experiences over time [6].

Women and girls residing in urban slum areas, such as those in Budanilakantha Municipality in Kathmandu, often face compounded vulnerabilities due to their socio-economic conditions, inadequate sanitation infrastructure, and lack of access to health education. These factors collectively impact their ability to manage menstruation safely and with dignity [7]. Various studies have identified socio-demographic characteristics including age, education, religion, maternal literacy, and income as significant determinants of menstrual hygiene practices [8–10].

Despite existing evidence from different parts of Nepal and South Asia, there is a dearth of localized data focusing on menstrual hygiene among women living in slum areas of Kathmandu Valley. This study was, therefore, conducted to identify the factors influencing menstrual hygiene practices among women aged 18–49 years residing in slum communities of Budanilakantha Municipality. Understanding these factors is essential for informing targeted interventions and policy measures aimed at improving menstrual health outcomes and ensuring gender equity in hygiene and sanitation.

Research question: What are the factors influencing menstrual hygiene management practices among women living in the slum areas of Budanilakantha Municipality, and what socio-demographic, behavioural, and environmental determinants shape these practices?

## Materials and Methods

### Methods

This community-based analytical cross-sectional study aimed to assess menstrual hygiene practices and associated factors among women living in selected slum areas of Budanilakantha Municipality, Kathmandu, Nepal. The study period was from July 25 to November 25 2022 in a semi-urban setting characterized by informal settlements with limited access to water, sanitation, and hygiene (WASH) services.

### Study Area

This study was conducted in selected wards of Budanilakantha Municipality, Kathmandu. Budhanilkantha Municipality is one of the urban municipalities in Kathmandu District, Bagmati Province, with a population of 177,557 according to the 2021 Census and an area of 34.8 km^2^. The municipality includes densely populated wards with informal settlements and limited access to safe water, sanitation, and adequate housing. The slum areas studied were characterized by overcrowding, shared sanitation facilities, and low socioeconomic conditions, which can hinder menstrual hygiene management.

### Study Participants

The study population consisted of women aged 18–49 years residing in slum areas of Budanilakantha Municipality. Only women who had menstruated within the past 12 months were included to avoid enrolling post-menopausal participants. Women were eligible for inclusion if they had lived in the selected slum areas for at least six months, were willing to participate, and provided written informed consent. In cases where the head of the household was unavailable during data collection, another responsible adult family member was consulted to facilitate access. Women were excluded if they did not reside in the designated slum areas, were unable to communicate due to serious health conditions, or declined to provide consent.

### Sampling Technique and Sample Size

The sample size was calculated using the single population proportion formula:


$$\begin{array}{c} n=\frac{z^{\text{2}}pq}{d^{\text{2}}} \end{array}$$

where n is the required sample size, z is the standard normal deviate corresponding to the desired confidence level, p is the estimated prevalence of good menstrual hygiene practice, q = 1 − p, and d is the margin of error. Assuming a prevalence of 39% for good menstrual hygiene practices [11] a 95% confidence level, and a 5% margin of error, the initial sample size was calculated. After adding 10% for non-response, the final sample size became 247.The study was conducted in four wards of Budanilakantha Municipality (Wards 4, 6, 8, and 10), which contain slum settlements. These wards were purposively selected as they represent all slum areas in the municipality. A total of 1,145 slum households were listed in collaboration with local health authorities, with 578 eligible women aged 18–49 years identified. The total sample size of 247 women was proportionally allocated across the selected wards using the Probability Proportionate to Size (PPS) technique Based on the number of eligible women in each ward, 25 respondents were selected out of 58 in Ward 4, 53 out of 123 in Ward 6, 153 out of 357 in Ward 8, and 16 out of 40 in Ward 10. Households were selected using systematic random sampling. The first household was chosen based on the bottle spin method, and if no eligible woman was found, the next consecutive household was selected. One eligible woman per household was interviewed.

### Data Collection Procedures

The questionnaire categorized menstrual absorbents as sanitary pads or reusable cloth, but did not capture details on cloth type, cleaning, drying, or storage practices. The questionnaire was developed following a review of the published literature on menstrual hygiene management (MHM) and water, sanitation, and hygiene (WASH), with particular emphasis on studies conducted in Nepal, including those from Dang and Lalitpur. The questionnaire was initially prepared in English, then translated into Nepali and back-translated to ensure accuracy and consistency. Key sections of the tool covered socio-demographic characteristics, knowledge and practices related to menstrual hygiene, access to WASH (Water, Sanitation, and Hygiene) facilities, and sociocultural restrictions surrounding menstruation. A pilot test was conducted among 10% of the total sample size in a similar slum area not included in the main study, and necessary adjustments were made based on the feedback received. Data collection was carried out through face-to-face interviews by trained enumerators. Field supervisors and the principal investigator performed daily reviews to ensure completeness and accuracy of the collected data.

### Variables

Independent variables included age, marital status, education, occupation, family income, ethnicity, religion, family structure, knowledge of menstruation, and cultural restrictions. The outcome variable was menstrual hygiene practices, assessed using a composite of reported behaviours (e.g., type of absorbent used, frequency of change, handwashing, and genital hygiene), and categorized into levels of practice rather than normative labels [1,2,12].

### Statistical Analysis

Data were entered into EpiData version 3.1 and analyzed using SPSS version 16. Descriptive statistics including frequencies, percentages, means, and standard deviations were used to summarize the data. Associations between menstrual hygiene practices and independent variables were examined using Chi-square tests. For tables with expected cell counts less than 5, categories were combined where appropriate to meet the assumptions of the Chi-square test; where this was not possible, results were interpreted cautiously. A p-value of less than 0.05 was considered statistically significant. Questionnaires with missing data on key outcome variables were excluded from the analysis, and only complete questionnaires were included in the final analysis

### Ethical Statement

Ethical approval for this study was obtained from the Institutional Review Committee of Nobel College, Pokhara University (Reference no. 01). Written informed consent was obtained from all participants prior to inclusion in the study; for non-literate participants, thumbprints were accepted as consent. Participants were informed of their right to withdraw at any time without penalty. Confidentiality and anonymity of participant information were maintained by using anonymized codes and secure storage of data in password-protected computers and locked cabinets. This study was conducted in accordance with the ethical principles of the Declaration of Helsinki (2013 revision) (28).

## Results

### Socio-demographic Characteristics of Respondents (n=247)

Table 1 summarizes the socio-demographic characteristics and menstrual hygiene practices of the 247 women included in the study. The majority of respondents (48.2%) were aged between 20 and 25 years, with a mean age of 24.8 years. Most participants were Hindu (69.7%), followed by Buddhists (27.9%). A significant proportion of respondents had only completed primary (34.4%) or secondary (25.1%) education, while just 5.7% had completed a bachelor’s degree or higher. Education categories were clarified to represent the highest completed level of formal education

**Table 1:** Socio-demographic characteristics and menstrual hygiene practices of respondents (n = 247).

Variable	Category	Frequency (n)	Percentage (%)
**Age Group (years)**	< 20	25	10.1
	20–25	119	48.2
	26–30	95	38.5
	30-49	8	3.2
**Religion**	Hindu	172	69.7
	Buddhist	69	27.9
	Christian	3	1.2
	Islam	3	1.2
**Education Level**	Unable to read/write Can read and write	9 43	3.6 17.4
	Primary	85	34.4
	Secondary	62	25.1
	Higher secondary	34	13.8
	Bachelor and above	14	5.7
			3.5
**Occupation**	Student	70	28.3
	Private service	57	23.1
	Housewife	30	12.1
	Labor	25	10.1
	Business	22	8.9
	Foreign Employment	7	2.8
	Govt Services	26	10.5
	Agriculture	10	4
**Type of Absorbent Used**	Sanitary pad	133	53.8
	Cloth	106	42.9
	Tampon	8	3.2
**Restriction During Menstruation**	Yes	235	95.1
	No	12	4.9
**Perceived as “Impure” During Menstruation**	Yes	128	57.1
	No	96	42.9

In terms of occupation, students comprised the largest group (28.3%), followed by women working in private services (23.1%) and housewives (12.1%). Regarding menstrual hygiene practices, 53.8% of the participants reported using sanitary pads during menstruation, while 42.9% used cloth and 3.2% used tampons. Here, ‘cloth’ refers to reusable fabric pieces traditionally used during menstruation, with varying levels of cleanliness Detailed information on cloth type, brand, absorbency, cleaning, drying, and storage practices was not collected. Nearly all respondents (95.1%) reported facing some form of restriction during menstruation. Cultural stigma was evident, as more than half of the participants (57.1%) reported being perceived as “impure” during menstruation. These findings reflect not only the socio-economic challenges but also the deep-rooted cultural barriers that influence menstrual hygiene practices among women living in slum areas. Nearly all respondents (95.1%) reported experiencing at least one sociocultural restriction during menstruation. Detailed categories of these restrictions are presented in Table 2

**Table 2:** Reinforcing factors related to menstrual hygiene among respondents.

Characteristic	Category	Frequency (n)	Percentage (%)
Food restriction (among those restricted, n = 235)	Yes	1	0.4
	No	234	99.6
Household work restriction (n = 235)	Yes	227	96.5
	No	8	3.5
Prayer restriction (n = 235)	Yes	1	0.4
	No	234	99.6
Celebration restriction (n = 235)	Yes	63	26.8
	No	172	73.2
Social problems during menstruation (n = 269)	Yes	224	81.8
	No	45	18.2
Untouchability followed in society (n = 224)	Yes	98	43.8
	No	126	56.2
Discrimination faced during menstruation (n = 224)	Yes	57	25.4
	No	167	74.6
Considered dirty or impure during menstruation (n = 224)	Yes	128	57.1
	No	96	42.9
Menstruation strictly followed family norms (n = 247)	Yes	198	80.2
	No	49	19.8
Menstruation taken normally in the family (n = 247)	Yes	49	19.8
	No	198	80.2

Table 2 presents the reinforcing sociocultural factors influencing menstrual hygiene practices among respondents. Among women who reported menstrual restrictions, the most common forms of restriction included restrictions related to household work (96.5%).and participation in celebrations (26.8%). Only a negligible number reported restrictions related to food (0.4%) and prayer (0.4%).

Social stigma surrounding menstruation was prevalent. More than four out of five respondents (81.8%) reported facing social problems during menstruation, and nearly half (43.8%) reported being subjected to untouchability practices. About one-quarter (25.4%) experienced direct discrimination during their menstrual cycle. Furthermore, 57.1% of participants reported being perceived as dirty or impure during menstruation.

In terms of family beliefs, 80.2% of women said that menstruation-related norms were strictly followed in their households, indicating the deep-rooted influence of traditional customs. Interestingly, only 19.8% of participants said menstruation was taken normally within their families, further underscoring the stigma and silence surrounding the topic. In this study, “menstruation-related norms” refer to culturally practiced restrictions and beliefs observed during menstruation in the study setting, including limitations on household work, participation in religious activities and social events, food-related restrictions, and perceptions of impurity associated with menstruation

Table 3 outlines the enabling factors and menstrual hygiene practices of the respondents. About two-thirds (65.6%) of women were aware of menstruation before its onset, and 88.7% reported receiving an adequate explanation when they first experienced it. The most common sources of menstrual information were family members (59.3%) and teachers (57.4%), followed by friends (30.7%) and social media (11.6%).

**Table 3:** Enabling factors and menstrual hygiene practices among respondents.

Characteristic	Category	Frequency (n)	Percentage (%)
Awareness of menstruation before onset (n = 247)	Yes	162	65.6
	No	85	34.4
Perceived adequacy of menstrual explanation (n = 247)	Adequate	219	88.7
	Inadequate	28	11.3
Source of information about menstruation (n = 394)	Family member	147	59.3
	Teacher	142	57.4
	Friends	76	30.7
	Social media	29	11.6
Type of absorbent used (n = 247)	Sanitary pad	133	53.8
	Cloth	106	42.9
	Tampon	8	3.2
Frequency of absorbent change (n-247)	Once per day	9	3.6
	2–3 times per day	143	57.9
	≥4 times per day	95	38.5
body washing during menstruation (n=247)	Daily	96	38.9
	Only when feeling dirty	66	26.7
	Only on fourth day	39	15.8
	Does not bathe	46	18.6
Non-disposable absorbent management (n = 37)	Washed and reused	34	91.9 (of 37)
	Thrown in dustbin	3	8.1 (of 37)
Disposable absorbent stored them (n = 210)	In toilet	181	86.2
	In bedroom	25	11.9
	Outside	4	1.9
Disposal method of disposable absorbent (n = 210)	Thrown in dustbin	130	61.9
	Burned	80	38.1

In terms of menstrual hygiene materials, 53.8% of participants used disposable sanitary pads refers to disposable menstrual pads, while reusable cloth pads were recorded separately under cloth., while 42.9% used cloth, and 3.2% used tampons. The majority of women (57.9%) reported changing their absorbent materials two to three times per day, and 38.5% changed them four or more times per day. Only 3.6% changed absorbents once daily.

Regarding hygiene behaviors, 66.8% of women used soap and water for handwashing during menstruation, whereas 33.2% used only water. Similarly, 82.6% cleaned their external genitalia with soap and water, while 17.4% used only water.

Bathing frequency varied: 38.9% bathed daily during menstruation, 26.7% bathed when feeling dirty, 15.8% bathed only on the fourth day, and 18.6% did not bathe during menstruation. Among women who used non-disposable absorbents, the majority (91.9%) reported washing and reusing them, while a small proportion (8.1%) discarded them.

In terms of storing used disposable absorbents before disposal, 86.2% kept them in toilets, 11.9% in bedrooms, and 1.9% outside. For disposal, 61.9% threw used absorbents into dustbins, while 38.1% burned them.

Table 4 presents the association between selected socio-demographic characteristics and awareness of menstruation before its onset. Education level was significantly associated with awareness (p = 0.043). Women who were unable to read and write had the lowest level of awareness (33.3%), whereas awareness increased with educational attainment, reaching 85.7% among women with a bachelor’s degree or higher. Monthly income was also significantly associated with awareness (p = 0.002). Women with a monthly income below NPR (Nepalese Rupees, the official currency of Nepal).)30,000 had the lowest awareness (50.0%), while awareness increased with income, reaching 78.5% among those earning more than NPR 90,000 per month. No statistically significant associations were observed for age, religion, ethnicity, marital status, occupation, or family type.

**Table 4:** Association between socio-demographic characteristics and awareness of menstruation before Its onset (n = 247).

Variable	Category	Aware (n, %)	Not Aware (n, %)	p-value
**Age**	< 20	20 (80.0%)	5 (20.0%)	0.858
	20–25	85 (71.4%)	34 (28.6%)	
	26–30	71 (74.7%)	24 (25.3%)	
	31-49	6 (75.0%)	2 (25.0%)	
**Religion**	Hindu	125 (72.6%)	47 (27.4%)	0.600
	Buddhist	52 (75.4%)	17 (24.6%)	
	Christian	3 (100.0%)	0 (0.0%)	
	Islam	2 (66.7%)	1 (33.3%)	
**Ethnicity**	Brahmin	22 (71.0%)	9 (29.0%)	0.938
	Chhetri/Thakuri	46 (75.4%)	15 (24.6%)	
	Madhesi	21 (80.8%)	5 (19.2%)	
	Muslim	2 (66.7%)	1 (33.3%)	
	Aadibasi/Janajati	56 (72.7%)	21 (27.3%)	
	Dalit	35 (71.4%)	14 (28.6%)	
**Marital Status**	Married	68 (79.1%)	18 (20.9%)	0.336
	Unmarried	112 (70.9%)	46 (29.1%)	
	Separated	1 (50.0%)	1 (50.0%)	
	Widowed	1 (100.0%)	0 (0.0%)	
**Education Level**	Unable to read/write	3 (33.3%)	6 (66.7%)	0.043
	Can read and write	34 (79.1%)	9 (20.9%)	
	Primary	64 (75.3%)	21 (24.7%)	
	Secondary	41 (66.1%)	21 (33.9%)	
	Higher Secondary	24 (70.6%)	10 (29.4%)	
	Bachelor and above	12 (85.7%)	2 (14.3%)	
**Occupation**	Housewife	25 (83.3%)	5 (16.7%)	0.220
	Agriculture	6 (60.0%)	4 (40.0%)	
	Government service	21 (80.8%)	5 (19.2%)	
	Private service	39 (68.4%)	18 (31.6%)	
	Business	18 (81.8%)	4 (18.2%)	
	Labor	16 (64.0%)	9 (36.0%)	
	Student	50 (71.4%)	20 (28.6%)	
	Foreign employment	7 (100.0%)	0 (0.0%)	
**Family Type**	Nuclear	59 (76.6%)	18 (23.4%)	0.990
	Joint	77 (67.5%)	37 (32.5%)	
	Extended	46 (82.1%)	10 (17.9%)	
**Monthly Income**	< 30,000 NPR	38 (50.0%)	38 (50.0%)	0.002
	30,000–60,000 NPR	67 (67.0%)	33 (33.0%)	
	61,000–90,000 NPR	33 (76.7%)	10 (23.3%)	
	> 90,000 NPR	22 (78.5%)	6 (21.5%)	

Table 5 presents the association between socio-demographic characteristics and the perceived adequacy of the explanation of menstruation. Monthly income was significantly associated with perceived adequacy of the explanation (p = 0.040). Women with a monthly income below 30,000 (NPR) had the lowest perceived adequacy (68.4%), whereas higher-income groups reported greater perceived adequacy, reaching 89.3% among women earning more than 90,000 NPR per month. No statistically significant associations were observed between perceived adequacy of the explanation and age, religion, ethnicity, marital status, education level, occupation, or family type.

**Table 5:** Association between socio-demographic characteristics and perceived adequacy of the explanation of menstruation (n = 247).

Variable	Category	Adequate Explanation n (%)	Inadequate Explanation n (%)	p-value
**Age**	< 20	21 (84.0%)	4 (16.0%)	0.054
	20–25	113 (95.0%)	6 (5.0%)	
	26–30	89 (93.7%)	6 (6.3%)	
	31-49	6 (75.0%)	2 (25.0%)	
**Religion**	Hindu	157 (91.3%)	15 (8.7%)	0.439
	Buddhist	62 (89.9%)	7 (10.1%)	
	Christian	2 (66.7%)	1 (33.3%)	
	Islam	3 (100.0%)	0 (0.0%)	
**Ethnicity**	Brahmin	30 (96.8%)	1 (3.2%)	0.288
	Chhetri/Thakuri	55 (90.2%)	6 (9.8%)	
	Madhesi	22 (84.6%)	4 (15.4%)	
	Muslim	3 (100.0%)	0 (0.0%)	
	Aadibasi/Janajati	69 (89.6%)	8 (10.4%)	
	Dalit	42 (85.7%)	7 (14.3%)	
**Marital Status**	Married	81 (94.2%)	5 (5.8%)	0.206
	Unmarried	148 (93.7%)	10 (6.3%)	
	Widowed	1 (100.0%)	0 (0.0%)	
	Separated	1 (50.0%)	1 (50.0%)	
**Education Status**	Unable to read/write	5 (55.6%)	4 (44.4%)	0.558
	Can read and write	33 (76.7%)	10 (23.3%)	
	Primary level	70 (82.4%)	15 (17.6%)	
	Secondary level	54 (87.1%)	8 (12.9%)	
	Higher secondary level	30 (88.2%)	4 (11.8%)	
	Bachelor and above	12 (85.7%)	2 (14.3%)	
**Occupation**	Housewife	29 (96.7%)	1 (3.3%)	0.551
	Agriculture	9 (90.0%)	1 (10.0%)	
	Government services	21 (80.8%)	5 (19.2%)	
	Private services	51 (89.5%)	6 (10.5%)	
	Business	18 (81.8%)	4 (18.2%)	
	Labor	19 (76.0%)	6 (24.0%)	
	Student	63 (90.0%)	7 (10.0%)	
	Foreign employment	7 (100.0%)	0 (0.0%)	
**Family Type**	Nuclear	69 (89.6%)	8 (10.4%)	0.294
	Joint	108 (94.7%)	6 (5.3%)	
	Extended	54 (96.4%)	2 (3.6%)	
**Monthly Income (NPR)**	< 30,000	52 (68.4%)	24 (31.6%)	**0.040** *
	30,000–60,000	89 (89.0%)	11 (11.0%)	
	61,000–90,000	37 (86.0%)	6 (14.0%)	
	> 90,000	25 (89.3%)	3 (10.7%)	

Table 6 presents the association between handwashing practices during menstruation and various socio-demographic characteristics of the respondents. Overall, 31.8% of participants reported using water only, while 68.2% used soap and water for hand hygiene during menstruation.

**Table 6:** Association between socio-demographic characteristics and hand wash practices followed by respondent (n = 247).

Variable	Category	Water Only n (%)	Soap and Water n (%)	p-value
**Age**	< 20	9 (36.0%)	16 (64.0%)	0.931
	20–25	38 (31.9%)	81 (68.1%)	
	26–30	33 (34.7%)	62 (65.3%)	
	31-49	2 (25.0%)	6 (75.0%)	
**Religion**	Hindu	52 (30.2%)	120 (69.8%)	0.212
	Buddhist	27 (39.1%)	42 (60.9%)	
	Christian	2 (66.7%)	1 (33.3%)	
	Islam	1 (50.0%)	1 (50.0%)	
**Ethnicity**	Brahmin	10 (32.3%)	21 (67.7%)	**0.001** *
	Chhetri/Thakuri	27 (44.3%)	34 (55.7%)	
	Madhesi	2 (7.7%)	24 (92.3%)	
	Muslim	2 (66.7%)	1 (33.3%)	
	Aadibasi/Janajati	31 (40.3%)	46 (59.7%)	
	Dalit	10 (20.4%)	39 (79.6%)	
**Marital Status**	Married	34 (39.5%)	52 (60.5%)	0.132
	Unmarried	47 (29.7%)	111 (70.3%)	
	Widowed	1 (100.0%)	0 (0.0%)	
	Separated	0 (0.0%)	2 (100.0%)	
**Education Status**	Unable to read/write	5 (55.6%)	4 (44.4%)	0.139
	Can read and write	15 (34.9%)	28 (65.1%)	
	Primary level	34 (40.0%)	51 (60.0%)	
	Secondary level	18 (29.0%)	44 (71.0%)	
	Higher secondary level	6 (17.6%)	28 (82.4%)	
	Bachelor and above	4 (28.6%)	10 (71.4%)	
**Occupation**	Housewife	9 (30.0%)	21 (70.0%)	**0.001** *
	Agriculture	2 (20.0%)	8 (80.0%)	
	Government services	8 (30.8%)	18 (69.2%)	
	Private services	18 (31.6%)	39 (68.4%)	
	Business	16 (72.7%)	6 (27.3%)	
	Labor	7 (28.0%)	18 (72.0%)	
	Student	17 (24.3%)	53 (75.7%)	
	Foreign employment	0 (0.0%)	7 (100.0%)	
**Family Type**	Nuclear	29 (37.7%)	48 (62.3%)	0.609
	Joint	35 (30.7%)	79 (69.3%)	
	Extended	18 (32.1%)	38 (67.9%)	
**Monthly Income (NPR)**	< 30,000	45 (59.2%)	31 (40.8%)	**0.001** *
	30,000–60,000	20 (20.0%)	80 (80.0%)	
	61,000–90,000	5 (11.6%)	38 (88.4%)	
	> 90,000	7 (25.0%)	21 (75.0%)	

There was a statistically significant association between handwashing practices and ethnicity (p = 0.001). A higher proportion of Madhesi (92.3%) and Dalit (79.6%) respondents reported using soap and water compared to other ethnic groups. Similarly, a significant association was observed with occupation (p = 0.001). All respondents in foreign employment and the majority of students (75.7%) reported using soap and water, whereas a higher proportion of businesswomen (72.7%) used only water.

Monthly household income was also significantly associated with handwashing practices (p = 0.001). Respondents with a monthly income below NPR 30,000 had the lowest soap usage (40.8%), whereas those with an income of NPR 61,000–90,000 reported the highest (88.4%).

No significant associations were found between handwashing practices and other variables such as age (p = 0.931), religion (p = 0.212), marital status (p = 0.132), educational status (p = 0.139), and family type (p = 0.609). However, a trend toward improved hygiene with higher education and income levels was noted.

Table 7 shows the association between respondents’ socio-demographic characteristics and their practices regarding the cleaning of external genitals during menstruation. The majority of participants reported cleaning with water only, while a smaller proportion used soap and water.

**Table 7:** Association between socio-demographic characteristics and cleaning of external genitals (n = 247).

Variable	Category	Water Only n (%)	Soap and Water n (%)	p-value
**Age**	< 20	9 (36.0%)	16 (64.0%)	0.931
	20–25	38 (31.9%)	81 (68.1%)	
	26–30	33 (34.7%)	62 (65.3%)	
	31-49	2 (25.0%)	6 (75.0%)	
**Religion**	Hindu	52 (30.2%)	120 (69.8%)	0.212
	Buddhist	27 (39.1%)	42 (60.9%)	
	Christian	2 (66.7%)	1 (33.3%)	
	Islam	1 (50.0%)	1 (50.0%)	
**Ethnicity**	Brahmin	10 (32.3%)	21 (67.7%)	**0.001** *
	Chhetri/Thakuri	27 (44.3%)	34 (55.7%)	
	Madhesi	2 (7.7%)	24 (92.3%)	
	Muslim	2 (66.7%)	1 (33.3%)	
	Aadibasi/Janajati	31 (40.3%)	46 (59.7%)	
	Dalit	10 (20.4%)	39 (79.6%)	
**Marital Status**	Married	34 (39.5%)	52 (60.5%)	0.132
	Unmarried	47 (29.7%)	111 (70.3%)	
	Widowed	1 (100.0%)	0 (0.0%)	
	Separated	0 (0.0%)	2 (100.0%)	
**Education Status**	Unable to read/write	5 (55.6%)	4 (44.4%)	0.139
	Can read and write	15 (34.9%)	28 (65.1%)	
	Primary level	34 (40.0%)	51 (60.0%)	
	Secondary level	18 (29.0%)	44 (71.0%)	
	Higher secondary level	6 (17.6%)	28 (82.4%)	
	Bachelor and above	4 (28.6%)	10 (71.4%)	
**Occupation**	Housewife	9 (30.0%)	21 (70.0%)	**0.001** *
	Agriculture	2 (20.0%)	8 (80.0%)	
	Government services	8 (30.8%)	18 (69.2%)	
	Private services	18 (31.6%)	39 (68.4%)	
	Business	16 (72.7%)	6 (27.3%)	
	Labor	7 (28.0%)	18 (72.0%)	
	Student	17 (24.3%)	53 (75.7%)	
	Foreign employment	0 (0.0%)	7 (100.0%)	
**Family Type**	Nuclear	29 (37.7%)	48 (62.3%)	0.609
	Joint	35 (30.7%)	79 (69.3%)	
	Extended	18 (32.1%)	38 (67.9%)	
**Monthly Income (NPR)**	< 30,000	45 (59.2%)	31 (40.8%)	**0.001** *
	30,000–60,000	20 (20.0%)	80 (80.0%)	
	61,000–90,000	5 (11.6%)	38 (88.4%)	
	> 90,000	7 (25.0%)	21 (75.0%)	

A statistically significant association was observed between family type and cleaning practices (p < 0.001). Among respondents from extended families, 26.8% reported using soap and water compared to only 5.2% in nuclear families and 7.0% in joint families.

No significant associations were observed with age (p = 0.08), religion (p = 0.501), ethnicity (p = 0.161), marital status (p = 0.667), education level (p = 0.141), occupation (p = 0.126), or monthly income (p = 0.207).

## Discussion

This study examined menstrual hygiene practices and their associated factors among women living in urban slum areas of Budanilakantha Municipality, Kathmandu. Four key findings emerged. First, higher educational attainment and monthly income were significantly associated with greater awareness of menstruation before its onset. Second, monthly income was also significantly associated with the perceived adequacy of menstrual education. Third, several socio-demographic characteristics were significantly associated with recommended menstrual hygiene practices, including handwashing and cleaning of the external genitalia during menstruation. Finally, although awareness of menstruation was relatively high, important gaps in menstrual hygiene practices persisted, particularly the continued use of reusable cloths and barriers related to water, sanitation, and prevailing socio-cultural restrictions. Maintaining menstrual hygiene, including regular bathing or washing the external genital area, is commonly recommended as part of good personal hygiene during menstruation Present study clearly distinguish our findings from those reported by Goddard and Sommer, who emphasized that urban slum populations often face infrastructural limitations that impede menstrual hygiene management (MHM) [7]. The study also revealed that cultural taboos and restrictions were widespread. About 95.1% of participants faced some form of restriction during menstruation, including. exclusion from household chores, celebrations, and religious practices. Moreover, 57.1% of respondents reported being considered “impure” during menstruation. The high level of menstrual stigma observed in this study is consistent with findings from other settings in South Asia, where cultural taboos and perceptions of impurity remain prevalent [9,13]. However, studies from non-slum or more urbanized populations have reported relatively lower levels of restriction and stigma, likely due to better access to education, resources, and awareness [6] This suggests that women living in slum areas may experience compounded challenges related to both socio-economic disadvantage and entrenched cultural norms However, in a study from urban slums in Karad, India, only 12.6% of adolescent girls used sanitary napkins while the majority relied on reusable cloths [10]. These differences may be attributed to varying levels of access to menstrual products, socio-economic status, and education.

Despite 65.6% of respondents reporting awareness of menstruation before menarche, the data indicate that knowledge does not always translate into practice. For instance, only 53.8% used sanitary pads, while 42.9% used cloth as their primary menstrual absorbent. Participants reported use of different menstrual absorbent materials, including sanitary pads, cloth, and tampons, however, this study did not assess the comparative hygienic effects of these products. This reflects findings from Deshpande et al., where only 60% of girls used sanitary pads and a majority lacked accurate knowledge of menstruation [14]. The mean age of respondents in this study was 24.8 years, and 53.8% reported using sanitary pads. This finding is comparable to the results from a study conducted in Kathmandu slums, where 65.6% of participants reported using sanitary pads and 96.1% practiced perineal cleaning during menstruation [15]. A significant association was observed between monthly income and awareness of menstruation before its onset (p = 0.002), as well as the perceived adequacy of information received (p = 0.04). This supports the findings from Habtegiorgis et al., who found that maternal education and household income significantly influenced good menstrual hygiene practices in Ethiopia [16]. Similarly, Bhusal et al. also observed a strong association between parental education and menstrual hygiene practices in Nepal [17]. The influence of ethnicity, occupation, and income on handwashing and cleaning practices was statistically significant (p < 0.05). These findings are supported by Chauhan et al., who reported that education level and socio-economic status are predictive of sanitary product usage and hygiene behavior [18].

This finding is consistent with global evidence highlighting that slum dwellers face compounded challenges related to poor sanitation, unsafe housing, and inadequate access to services, which impact menstrual hygiene practices and overall health [19].

While this study provides a comprehensive overview of menstrual hygiene practices in urban slums, further attention is warranted on enabling environmental factors and policy-level gaps. The findings indicate that although a majority of women used soap and water for hygiene, only 38.9% bathed daily during menstruation, and a participant (18.6%) did not bathe at all. These figures reflect broader WASH infrastructure deficits in slum communities. According to WHO and UNICEF, the availability of private, safe, and clean spaces for bathing is essential for (MHM), yet remains lacking in informal settlements globally [20]. Studies from sub-Saharan Africa and South Asia have emphasized how shared or unsafe toilets and bathing areas prevent women from practicing MHM effectively, often leading to embarrassment and avoidance of hygiene routines [21,22]. Nearly 82% of women in this study reported facing social problems, and more than half felt perceived as “impure.” While many studies quantify stigma, recent work by Tellier et al. (2021) argues that menstrual stigma contributes to long-term mental health consequences, including shame, low self-esteem, and social isolation, particularly among marginalized women and girls [23]. These effects are often compounded in slum settings where the intersection of poverty and gender inequity limits resilience and access to mental health support. Disposal practices of menstrual products also deserve greater attention. The temporary storage of used absorbents within toilet or bathroom areas, as reported by the majority of participants, may have important hygiene and environmental implications in densely populated slum settings. Inadequate waste containment, limited ventilation, and shared sanitation facilities can increase the risk of odour microbial contamination, and strain on already overburdened sanitation systems. These challenges may discourage optimal menstrual hygiene practices and highlight the need for improved waste management solutions tailored to low-resource urban environments.61.9% threw used absorbents into dustbins, while 38.1% burned them. Open burning of sanitary waste, especially in dense urban settings, has environmental and health implications. A review by Van Eijk et al. (2019) highlighted that open burning of used menstrual absorbents may contribute to local air pollution and unpleasant odors, while disposal in unmanaged waste systems may add to environmental contamination and sanitation challenges in densely populated settlements. The discussion continues to note that improper disposal methods are commonly reported in low- and middle-income settings [12]. The lack of a safe and culturally appropriate waste management system can discourage the use of disposable pads, further reinforcing cloth usage and suboptimal hygiene practices. This study has several limitations that should be considered when interpreting the findings. First, the study did not differentiate between types of reusable cloths or assess their cleaning and storage practices, which may influence menstrual hygiene practices. Second, although the questionnaire was developed based on an extensive review of the literature and adapted to the local context, it was not based on existing validated menstrual health instruments, such as the Demographic and Health Surveys (DHS), Multiple Indicator Cluster Surveys (MICS), or other validated menstrual health measures. This approach was chosen because existing instruments do not comprehensively capture the socio-environmental challenges, cultural restrictions, and contextual factors experienced by women living in urban slum settings in Nepal. Nevertheless, the use of a study-specific questionnaire may limit direct comparison with studies using standardized instruments. Finally, the findings may have limited generalizability to other settings within Nepal and beyond, particularly where menstrual experiences, social norms, and access to resources differ.

In Nepal, menstrual health has received increasing policy attention in recent years; however, implementation of these policies remains inconsistent, particularly in marginalized and informal urban settlements. Thus, despite growing policy recognition, important gaps persist in translating policy commitments into effective programmes and equitable access to menstrual health services [24]. A gender-equity-focused approach must extend beyond adolescent girls in schools to include community-based interventions for adult women, especially in socioeconomically disadvantaged urban populations. A recent policy analysis by Mahon et al. emphasized that integrating menstrual health with broader sexual and reproductive health programmes and urban planning may support the long-term sustainability and implementation of menstrual health intervention [25].

The role of digital media and technology also represents an emerging opportunity. Only 11.6% of respondents in this study reported social media as a source of menstrual knowledge, where as a study in Bandung Regency demonstrated that a targeted intervention significantly improved menstrual hygiene knowledge, with a mean difference-in-differences (DiD) effect of 12.5 points (95% CI: 10.2–14.7; p < 0.001) compared to the control group [26]. Scaling up these tools could improve health literacy and challenge stigma where traditional communication channels fall short.

Overall, this study reinforces the need for comprehensive MHM interventions that include awareness programs, improvement in WASH infrastructure, and efforts to challenge harmful social norms. The findings emphasize that MHM is not only a health issue but also a matter of dignity, equity, and rights for women and girls living in marginalized communities. This study did not capture detailed information on menstrual waste handling practices, such as whether used absorbents were wrapped prior to storage or the duration of storage before disposal, which may influence hygiene and sanitation outcomes.

## Conclusions and Recommendations

A complex interplay of socioeconomic factors, cultural restrictions, and access to resources shapes menstrual hygiene practices among women in the slum areas of Budanilakantha Municipality. Despite moderate awareness and adoption of hygienic practices such as using sanitary pads and washing with soap and water, the pervasive cultural stigma and restrictions faced by nearly all women underscore the urgent need for interventions that address not only knowledge and infrastructure but also social norms and taboos. Enhancing menstrual health in such marginalized urban settings requires comprehensive strategies combining education, improved WASH facilities, stigma reduction, and inclusive policies that empower women and promote dignity and equity. Programs promoting menstrual health should integrate educational, infrastructural, and social support interventions to address multifactorial barriers faced by women living in slum areas. Although sanitary pads were regularly available at the local health post, structured MHM counselling or menstrual health strategies should address broader social determinants of health, including economic disadvantage, educational opportunities, and access to WASH services, alongside efforts to reduce stigma and strengthen community-based support.

## Supporting information

S1 File Revised_Deidentified_Dataset. (XLSX)

S2 File Questionnaire. (PDF)
