# Supplementary material for: Factors affecting menstrual hygiene among women living in slum areas of Budanilakantha Municipality, Kathmandu
Source: PLoS One. 2026 Aug 7;21(8):e0336321. doi: 10.1371/journal.pone.0336321 (PMC13450778; doi:10.1371/journal.pone.0336321)
Supplement: Supplementary file 1 [file pone.0336321.s002.pdf]

ANNEX-III: QUESTIONNAIRES

Section 1: Predisposing Characteristics

**Questions**	**Response Options**
What is your age?	………….
What is your religion?	Hindu<br>Muslim<br>Buddhist<br>Christian<br>Others (………
What is your ethnicity?	Brahmin<br>Chhetri<br>Madhesi<br>Muslim<br>Janajati/Aadibasi<br>Others (……….)
What is your marital status?	Married<br>Unmarried<br>Widowed<br>Separated<br>Divorced
What is your educational status	Unable to read and write<br>Can read and write<br>Primary level<br>Secondary level<br>Higher secondary<br>Bachelor and above
What is your occupation?	Agriculture

	Housewife<br>Labor<br>Foreign Employment<br>Public Service<br>Private<br>Business<br>Others (…………)
What is your family type?	Nuclear<br>Joint<br>Extended
Family Income (Yearly)	…………

Section 2: Reinforcing factor

**Question**	**Response options**
Awareness about menstruation before occurrence	yes<br>no
Perceived adequacy of the explanation of menstruation	Adequate information<br>Inadequate information
Source of Information	Family<br>Teacher<br>Friends<br>Social Media
Type of Absorbent usage	Pad<br>Piece of cloth<br>Menstrual cup<br>Tampon
Taking bath during menstruation	Only on 4th day<br>Daily<br>When I feel dirty<br>Doesn’t bath
Number of times absorbent changed	Once<br>Twice or thrice<br>Four times or more<br>none
Proper Handwashing Practice followed by respondent	Handwash with water<br>Handwash with soap and water<br>Doesn’t wash hand

Cleaning of external genitalia	with water<br>with soap and water<br>Doesn’t clean
Non-disposable absorbent management	Yes<br>No
Disposable absorbent keeping behavior	Washing dishes<br>Washing clothes<br>Brooming<br>Others…
Disposable absorbent management	Yes<br>No
What type of mental problems do you face during menstruation	Mood Swings<br>Irritability<br>Anxiety<br>Other Problems
Are there any social problems regarding menstruation in your society?	Untouchability followed in society<br>Discrimination during the menstruation<br>Considered "dirty" and "Impure"
Family perception during menstruation	Menstruation taken strictly following norms<br>Menstruation taken normally
Physical Problems during menstruation	Primary dysmenorrhea<br>Secondary dysmenorrhea
Restrictions followed	Avoid Celebrations and festivals<br>Avoid Certain Foods<br>Avoid House hold Works<br>Avoid Prayers
